# How Did School Meal Access Change during the COVID-19 Pandemic? A Two-Step Floating Catchment Area Analysis of a Large Metropolitan Area

**DOI:** 10.3390/ijerph182111350

**Published:** 2021-10-28

**Authors:** Jason Jabbari, Yung Chun, Pranav Nandan, Laura McDermott, Tyler Frank, Sarah Moreland-Russell, Dan Ferris, Stephen Roll

**Affiliations:** Social Policy Institute, Brown School, Washington University in St. Louis, One Brookings Drive, St. Louis, MO 63130, USA; yungchun@wustl.edu (Y.C.); pnandan@wustl.edu (P.N.); laura.mcdermott@wustl.edu (L.M.); tylerfrank@wustl.edu (T.F.); smoreland-russell@wustl.edu (S.M.-R.); dan.ferris@wustl.edu (D.F.); stephen.roll@wustl.edu (S.R.)

**Keywords:** COVID-19, school meal access, summer meal programs, two-step floating catchment area analyses

## Abstract

SARS-CoV-2 (COVID-19) resulted in school closures and contingencies across the U.S. that limited access to school meals for students. While some schools attempted to provide alternative meal access points where students or parents could pick up meals, many students—especially those in low-income households—lacked adequate transportation to these access points. Thus, physical proximity to meal access points was particularly important during the pandemic. In this study, we explore how school meal access changed during the COVID-19 pandemic, especially as it relates to race/ethnicity and socio-economic status. Taking into account both the “supply” (meal access points) and the “demand” (low-income students) for free meals, we employed a two-step floating catchment area analysis to compare meal accessibility in St. Louis, Missouri before and during the pandemic in the spring and summer of 2019 and 2020. Overall, while school meal access decreased during the spring of 2020 during the early months of the pandemic, it increased during the summer of 2020. Moreover, increased access was greatest in low-income areas and areas with a higher proportion of Black residents. Thus, continuing new policies that expanded access to school meals—especially for summer meal programs—could lead to positive long-term impacts on children’s health and well-being.

## 1. Introduction

On 11 March 2020, the COVID-19 was declared a global pandemic by the World Health Organization (Geneva, Switzerland). Two days later, a national emergency was declared in the U.S. After this declaration, most kindergarten through 12th grade schools closed their physical doors [1]. While schools were able to offer instruction virtually, these school closures made it more difficult to serve school meals, which can promote students’ health, development, and achievement [2]. Moreover, the disruptions in school meal access posed disproportionate risks to students from low-income families. For many of these students, the free and reduced-price meals they receive from school make up the majority of their diet [3]. The physical closure of schools thus created a barrier to accessing a basic human need for over 30 million students who participate in the national school lunch and breakfast programs in the U.S. [4].

Many school leaders and policy makers attempted to overcome this barrier by providing students with alternative options for accessing free meals during the pandemic [5]. They did so by utilizing federal waivers and flexibilities to introduce and expand meal locations during the spring of 2020. However, as many students lacked adequate transportation and most alternative access plans required meals to be picked up at these locations [5], physical proximity to these meal access points is of primary importance for students’ health and well-being. Furthermore, proximity to these meal access points may be unevenly distributed across geographic locations, potentially creating a new source of geographic inequality. Despite the substantial disruptions in meal access during COVID-19 and the associated risks to students from low-income families who may lack other nutrition options, there have been few studies examining this issue. The objective of this study is to understand how meal accessibility changed during COVID-19. The research questions motivating this study are:

1. How did school meal accessibility change during COVID-19?
2. How do changes in meal accessibility during COVID-19 relate to race/ethnicity and socioeconomic status?

**Figure 1:**
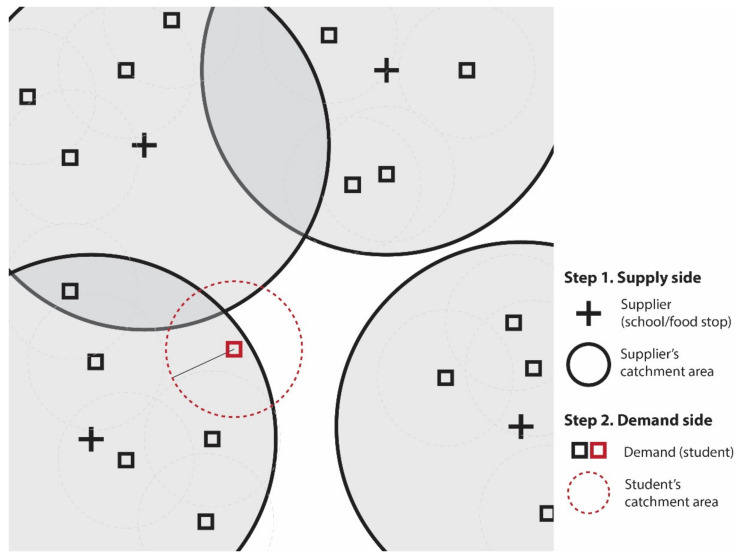
Study conceptualization of supply and demand dynamics using a two-step floating catchment area analysis.

To conduct this analysis, we analyzed physical proximity to meal access points in 2019 and 2020 in St. Louis, Missouri. We considered both the “supply” of free meals in the region as well as the “demand” for them by employing a two-step floating catchment area analysis [6]—an approach that accounts for both the number of meal access points within a given geographic region (i.e., supply) and the number of families living below the federal poverty level (i.e., demand). An illustration of this analysis can be seen in Figure 1.

We find that while meal access during the spring semester of 2020 (May 2020) was substantially lower when compared to the previous spring semester (May 2019), meal access during the summer of 2020 was substantially higher when compared to the previous summer. Additionally, increased access was most prevalent in low-income areas and areas with a higher proportion of Black residents.

## 2. Literature Review

### 2.1. Child Food Insecurity and School Meals

Prior to COVID-19, approximately 14% of households with children experienced food insecurity [7], which can have a negative effect on children’s academic performance, physical health, and social skills [8]. As participation in school meal programs decreases food insecurity and increases nutrition [9], it is unsurprising that school meals are associated with improved health [10], behavior [11], and academic achievement [2,12]. Nearly 30 million students participate in the National School Lunch Program and nearly 15 million students participate in the School Breakfast Program [13]. Each program is administered within the states and managed by the U.S. Department of Agriculture. Children with household incomes below 130% of the federal poverty level qualify for free meals, as do those from households receiving the Supplemental Nutrition Assistance Program (SNAP) and Temporary Assistance for Needy Families (TANF); children with household incomes below 185% of the federal poverty level qualify for reduced-price meals.

Moreover, as many students receiving free and reduced-priced meals during the school year could likely benefit from these offerings during the summer months, summer meals have become an important topic for policy-makers and other stakeholders interested in alleviating child food insecurity. The Summer Meals Program is commonly administered through the Seamless Summer Option and the Summer Food Service Program—where schools, as well as camps, nonprofit organizations, and other government agencies, can receive reimbursements for serving summer meals. Huang et al. found increased food insecurity in June and July for families that participated in free and reduced-price lunch programs during the school year [14], while Khun demonstrated that summer meals may help alleviate cyclical food insecurity [15]. Nevertheless, without mandatory attendance and a number of program restrictions, it is difficult for many low-income students to access summer meals. Only 14.1% of children who received free or reduced-priced lunches during the 2017–2018 school year received free or reduced-priced lunches during the following summer [16]. As spring 2020 school closings resemble a typical summer in terms of meal offerings, it is unsurprising that recent research shows increased food insecurity for families with children during the COVID-19 pandemic [17].

### 2.2. Access to School Meals

Recently, studies have incorporated detailed spatial analyses in the assessment of geographic opportunity and food security. For example, Miller examined driving time to summer meal sites for households that filled out the California Health Interview Survey [18]. Using a gravity model that takes into account both the supply of summer meals and the demand for them, this research found that geographic accessibility was associated with decreased probability of very low food insecurity among households with young children, as well as households that lived in suburban, town, or rural areas. In another study in Texas, Wilkerson et al. found that urban areas were more likely to have summer meal access points [19]. More recently, using meal distribution sites during the spring 2020 semester across Los Angeles, Houston, Chicago, and New York City, McLoughlin et al. demonstrated that while there were some gaps across all three districts, meal distribution sites were often in larger areas, higher poverty areas, and areas with greater proportions of minorities [5].

### 2.3. COVID-19 and School Meal Policies

In response to COVID-19 and the nationwide school closures that followed, the federal government passed the Families First Coronavirus Response Act (FFCRA) and the Coronavirus Aid, Relief, and Economic Security (CARES) Act. These efforts provided billions in funding for additional food supports through existing programs like SNAP and new programs like the Pandemic Electronic Benefit Transfer program (P-EBT), as well as flexibilities meant to help increase access to existing nutrition supports. Most relevant to our study, the FFCRA gave the USDA authority to grant waivers in order to help schools and other community organizations provide meals and snacks during COVID-19-related school closures [20,21]. These waivers are open to all states and allow for increased flexibilities in where, when, and how meals are served during the COVID-19 pandemic through the Seamless Summer Option (SSO) and the Summer Food Service Program (SFSP) [22]. These flexibilities include the following:

- An SSO/SFSP Area Eligibility Waiver that waives the summer meal program requirement limiting “open site” meal service to areas where a minimum of half of the children in the area are from low-income households;
- A Meal Time Waiver that allows for meals to be served outside standard meal times;
- A Non-congregate Feeding Waiver that allows meals to be provided outside of typical group settings; and
- A Nationwide Parent/Guardian Meal Pickup Waiver allowing parents and/or guardians to pick up meals for their children without the student being present (adopted in 41 states).

## 3. Methods

### 3.1. Sample Characteristics

This study focuses on St. Louis, Missouri, which includes both St. Louis City and St. Louis County. St. Louis is uniquely situated for a geographic exploration of this type, as it is sharply divided across an urban core (St. Louis City) and surrounding suburbs (St. Louis County). As most of the studies that explore geographic opportunity in relation to food access have focused on either urban cores [23] or rural areas [24], food accessibility in suburban areas remains largely unknown. This gap in the research is especially problematic when considering that many social services have been unable to keep up with dramatic rises in suburban poverty [25].

As we analyze St. Louis’s response to student meal access during COVID-19, it is important to understand the nature of poverty and food insecurity that existed in St. Louis and the state of Missouri prior to the pandemic. While estimates from the National Center of Education Statistics (NCES) demonstrate that the percentage of students in Missouri eligible for free or reduced-priced lunches was only slightly higher than the national average (53% compared to 52%), Missouri has one of the lowest rates of summer meal participation in the country. With only 8.5% of children who receive free or reduced-price lunches during the regular school year also receiving free or reduced-price lunches during summer, Missouri ranks 44th in terms of summer meal participation [26]. In terms of poverty, St. Louis City has a poverty rate of 25%, which differs greatly across race and ethnicity. Despite making up 45% of the City’s population, Black individuals make up 64% of those living in poverty [27]. Conversely, St. Louis County has a poverty rate of just 10% [27]. Similarly, this rate also differs greatly across race and ethnicity; despite making up just 24% of the County’s population, Black individuals make up 45% of those living in poverty [27].

When considering the local policy context during COVID-19, Missouri public schools closed on 20 March 2020, and on 6 April, stay-at-home orders for St. Louis City and County were put in place that lasted until 18 May [28]. In response to COVID-19, Missouri has implemented many of the core federal policy provisions previously mentioned in order to maintain student meal access during the pandemic. These include the SFSP/SSO Area Eligibility Waiver, the Meal Time Waiver, the Non-congregate Feeding Waiver, and the Nationwide Parent/Guardian Meal Pickup Waiver. In doing so, meals at Seamless Summer Option and Summer Food Service Program locations were provided during spring and summer 2020 at no cost to children 18 and under, regardless of their household income [29]. Additionally, flexibilities allow for districts to (a) serve meals at unrestricted times; (b) allow meals to be eaten off site; and (c) allow meals to be picked up by other family members. Together, these flexibilities allowed for less restricted access to meals during the COVID-19 pandemic, while simultaneously limiting opportunities for disease transmission.

### 3.2. Analytic Approach

We estimated accessibility to free, school-sponsored meals before and during the COVID-19 pandemic in three ways. First, using the average number of meal access points, we demonstrated how meal accessibility before and during COVID-19 relates to both family poverty and supermarket access. Second, using Euclidean (or straight-line) distances, we demonstrated how meal accessibility relates to free and reduced-price lunch status. Finally, using a gravity-based model that considers both family poverty and the number of meal access points, we demonstrated how meal accessibility changed during COVID-19 and how this change relates to urbanicity and racial/ethnic composition.

Measuring geographic food access can rely on measures of either proximity or density [30]. The proximity approach estimates the presence of spatial dependence between suppliers (e.g., grocery stores) and demand (e.g., shoppers). The proximity approach, however, often neglects the fact that the number of accessible suppliers can be as significant as the distance to the nearest supplier. By contrast, a density approach estimates how the intensity of accessible suppliers varies over a geographic area. The density approach typically sets a boundary (e.g., ZIP code area, census tract, etc.) and measures density by dividing the number of suppliers in an area by its population or its land area. Nevertheless, the density approach can be prone to bias, as it tends to focus on either the supply or the demand, but not both. The density approach is also vulnerable to the Modifiable Arial Unit Problem (MAUP), which highlights the potential of bias in the arbitrary delineation of a spatial unit’s boundaries [31].

To overcome the problems with both proximity- and density-based approaches, we employed a gravity-based accessibility approach known as a two-step floating catchment area (2SFCA) analysis [6]. This approach effectively combines the proximity and the density approaches and has been used to investigate food access inequities for Black individuals in Columbus, Ohio [31] and the geographical accessibility to SNAP-authorized retailers in Arkansas [32].

In this study, the catchment area is the geographic area from which families are able to obtain food from a meal access point (supply) for their children (demand). The model consists of two steps. First, it measures each supplier’s service intensity within a defined geographic boundary in relation to the surrounding potential beneficiaries (families in poverty).

(1) $$R_{j}=\frac{S_{j}}{\sum_{k\in \left\{d_{kj}\leq d_{o}\right\}}P_{k}}$$

The service intensity of a food supplier j $\left(R_{j}\right)$ is the function of the number of suppliers $\left(S_{j}\right)$ and the number of beneficiaries in a census block group $k\left(P_{k}\right)$ where the distance between its population center and the supplier $\left(d_{kj}\right)$ is not greater than a determined bandwidth $\left(d_{o}\right)$. We set $S_{j}$ as 1 for every meal access point. For beneficiaries $k\left(P_{k}\right)$, we used the number of families in poverty. We used a 1-mile bandwidth, following the USDA’s food access definition for urban areas [33]. The second step of this analysis sums the estimated ratios “floating” around each demand location (i.e., census block groups). The accessibility score for a census block group i ($A_{i})$ is as follows:

(2) $$A_{i}=\sum_{j\in \left\{d_{ij}\leq d_{o}\right\}}R_{j}$$

(3) $$=\sum_{j\in \left\{d_{ij}\leq d_{o}\right\}}\frac{S_{j}}{\sum_{k\in \left\{d_{kj}\leq d_{o}\right\}}P_{k}}$$

We used ArcMap v. 10.6.1 (ESRI, Redlands, CA, USA) to conduct the analysis and QGIS (an open source software) to produce all visualizations.

### 3.3. Data and Measures

The locations of meal access points in 2020 were downloaded from Missouri’s Coronavirus GIS Hub [34]. Meal access points for 2019 were obtained via request from the Missouri Department of Elementary and Secondary Education (DESE) and via the Sunshine Law and a public records request from the Missouri Department of Health and Senior Services. DESE Seamless Summer Option data were already geocoded. To geocode Summer Food Service Program data from the Department of Health and Senior Services, schools from the Department of Health and Senior Services dataset were merged with the DESE school directory dataset, and data points that were not merged (e.g., those at community parks or town centers) were manually geocoded using Google Maps. Shapefiles for school districts, school buildings, census block groups, and census tracts were downloaded from the Missouri Spatial Data Information Service archive [35]. Spring 2019 meal access points included any locations that served FRP meals during May. Spring 2020 meal access points included any locations that served FRP meals during May as well; in order to maintain consistency, spring 2020 meal access points were recorded during the first week of May while the spring semester was still in session. Summer 2019 meal access points included any locations that served meals during the summer school session (late May through July). Summer 2020 meal access points included any locations that served meals during the first week of July.

We obtained family poverty data from the 5-year estimates of the 2018 American Community Survey. We downloaded population-weighted centroids for census block groups from the U.S. Census Bureau. Using the Census’s continuous measure of family poverty, we also created a binary measure of family low-income (FLI) status that follows a common formula used by the Food Access Research Atlas [36]. Here, census tracts with poverty rates of at least 20% for families with children or a median income for families with children at or below 80% of the metropolitan area or state median income were assigned a family low-income status. It is important to note that our measure of local demand (i.e., the proportion of families before the poverty line) is not an exact match to the income profile of households who can access free or reduced price school meals used during a typical school year, as children living in households up to 130% of the Federal Poverty Level (FPL) can receive free meals during school year, while children living in households up to 185% of the FPL can receive reduced-price meals. Nevertheless, our definition of family low-income status is commonly used in food and nutrition research and allows us to focus on a population that was disproportionately impacted by the COVID-19 pandemic [37]. Given this, our measure of family low-income status is appropriate for this analysis.

We also used a measure that converges income status with food access, which was created by the Food Access Research Atlas. Combining low-income (LI) designations identical to our FLI designation with low-access (LA) designations, the Food Access Research Atlas created a status that measures the overlap of LI and LA census tracts—known as LILA census tracts [36]. LA status is determined by proximity to the nearest supermarket and accounts for differences in urban and rural areas. As noted by Rahkosky and Snyder, “a tract is classified as low access if at least 500 people or 30 percent of residents live more than 1 mile from a supermarket in urban areas” [38].

We obtained free or reduced-price lunch (FRPL) rates from DESE and the Prime Center at St. Louis University [39]. We categorized FRPL rates based on Community Eligibility Provision (CEP) criteria. DESE provides schools the option to offer free meals to all students in high-poverty schools through the CEP [40], a mechanism established by Congress in the Healthy-Hunger Free Kids Act of 2010. As previous research demonstrated that a greater proportion of CEP schools adopt more innovative meal service models in Missouri, such as grab-and-go meals [41], CEP-participating schools may be better equipped to provide food during the pandemic. In order to be eligible for CEP, schools must have at least 40% of identified students who are certified for free or reduced-price school meals without the use of a household application (e.g., directly certified with Supplemental Nutrition Assistance Program (SNAP), Temporary Assistance for Needy Families (TANF), Food Distribution Program on Indian Reservations (FDPIR), or based on status as migrant youth, homeless, foster child, Head Start, etc.) [42]. The percentage of students that qualify for FRPL is then multiplied by a factor of 1.6 to determine a free-meal-claiming percentage for the school. Thus, schools that have 62.5% of identified students that qualify for FRPL can have 100% free meal claims. Without available data on identified student percentages, we used free or reduced-price lunch eligibility rates to approximate CEP.

Additionally, we obtained racial composition and poverty concentration data from the 5-year estimates of the 2018 American Community Survey. Racial composition was modeled continuously based on the percentage of individuals who identified as Black in each census tract. Poverty concentration was also modeled continuously based on the total number of families in poverty in each census tract.

## 4. Results

### 4.1. An Overview of Food Access and School Meals in St. Louis, Missouri

**Figure 2:**
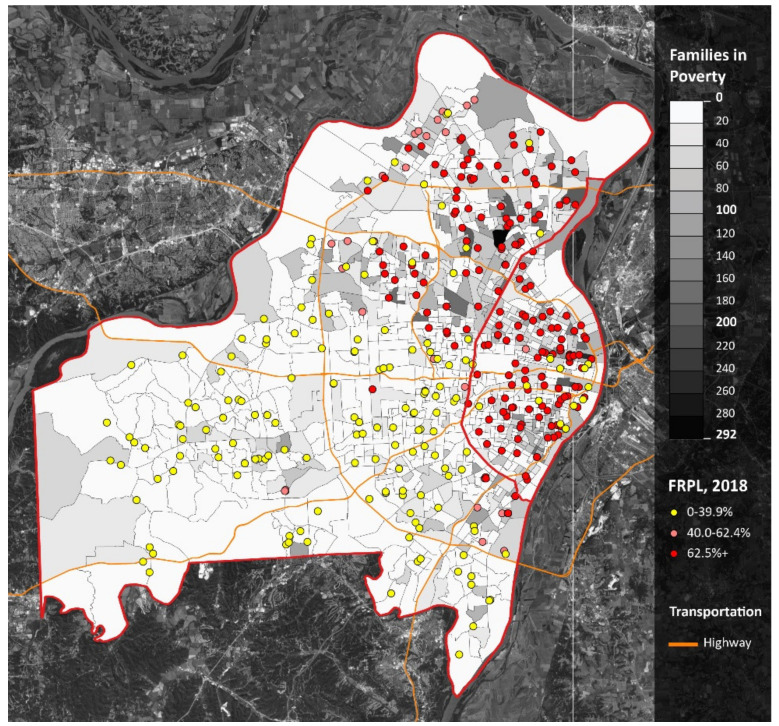
Free and reduced-price lunch in St. Louis, MO. Notes: St. Louis City is the area east of the red line. St. Louis County is the area west of the red line. Dots represent free or reduced- price Lunch categories from the 2018–2019 school year. Orange lines represent major highways. Darker shades of gray represent larger numbers of families in poverty.

Nearly half (46%) of the census tracts in St. Louis City and County are considered low-income—mirroring the state of Missouri (Table 1). Yet, due to the relative density of its residents and the proximity to grocery stores, only 10% of the census tracts are considered low-income, low-access (LILA). Over half (53%) of the public schools in St. Louis serve a student population where at least 62.5% of students qualify for free or reduced-price lunch, which can allow schools to serve free and reduced-price meals to all of their students under the Community Eligibility Provision. For schools located in low-income census tracts, the average proportion of students who are eligible for FRPL is 86%, compared to 39% for schools that are not located in low-income census tracts. Similarly, for schools located in LILA census tracts, the average proportion of students who are eligible for FRPL is 79%, compared to 57% for schools that are not located in LILA census tracts. As seen in Figure 2, the majority of schools with the highest proportions of students that are eligible for FRPL are in St. Louis City and the northern suburbs in St. Louis County. These are also areas with larger numbers of families in poverty.

### 4.2. How Did Meal Accessibility Change during COVID-19?

Unsurprisingly, the average number of meal access points during the regular school year prior to COVID-19 (spring 2019) is greater than one in all census tract categories (Table 2), as census tracts typically contain a school that serves free or reduced-price meals to students. Moreover, when considering summer meals prior to COVID-19 (i.e., summer meals served in 2019), the average number of meal access points was much larger in low-income census tracts when compared to higher income census tracts: 0.35 compared to 0.13. Nevertheless, in spring 2020 (during COVID-19), the average number of meal access points in low-income census tracts was only slightly less than the average number of meal access points prior to COVID-19: 0.94 in spring 2020 compared to 1.08 in spring 2019. In theory, students in low-income areas had similar opportunities to receive free meals during COVID-19 (spring 2020) as they did prior to COVID-19 (spring 2019). This, however, was not the case in higher income census tracts. Rather, the average number of meal access points was substantially lower during COVID-19: 0.45 in the spring of 2020 compared to 1.22 in the spring of 2019. In the summer of 2020, the average number of meal access points roughly quintupled to 1.76 in low-income census tracts when compared to the previous summer. Again, this was not the case in higher income census tracts where the average number of meal access points only slightly increased from summer 2019 to summer 2020. When considering income and food access together, a similar trend emerged: the average number of meal access points in LILA census tracts substantially increased during COVID-19. In fact, there were more food access points in spring 2020 than there were in spring 2019 within LILA census tracts.

When we consider school districts, we see similar trends. Meal access points within school districts decreased from spring 2019 to spring 2020 yet increased from summer 2019 to summer 2020 (Table 3). Moreover, when focusing on absolute differences, for schools with higher proportions of students that qualify for FRPL, the decrease from spring 2019 to spring 2020 was much smaller, while the increase from summer 2019 to summer 2020 was much larger. For example, among schools where at least 62.5% of the students qualify for FRPL, meal access points dropped from 21.0 in spring 2019 to 19.4 in spring 2020, compared to schools where less than 40% of the students qualify for FRPL, which experienced a drop from 12.5 in spring 2019 to 0.69 in spring 2020. Conversely, among schools where at least 62.5% of the students qualify for FRPL, meal access points increased from 6.8 in summer 2019 to 32.5 in summer 2020, a substantial increase compared to schools where less than 40% of the students qualify for FRPL, which experienced an increase from 0.10 in spring 2019 to 1.60 in spring 2020.

Similarly, average distances to the closest meal access points from schools increased from spring 2019 to spring 2020 yet decreased from summer 2019 to summer 2020. We also saw much shorter distances in schools with higher proportions of students qualifying for FRPL. For example, the distance to the closest meal access point in spring 2020 was 0.71 miles in schools where over 62.5% of the students qualify for FRPL, compared to 4.64 miles in schools where less than 40% of the students qualify for FRPL.

Finally, when considering both supply and demand for meal access, measures from our 2SFCA analyses corroborate many of the previously mentioned trends. First, there was a considerable drop-off in accessibility in the regular school year during COVID-19; meal accessibility in spring 2020 was roughly one-third the accessibility in spring 2019 (Table 4).

**Figure 3:**
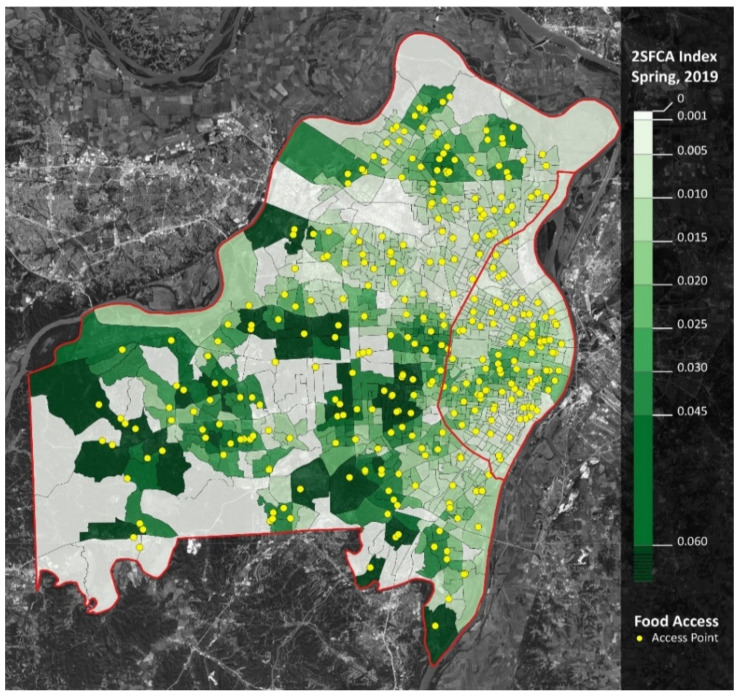
Meal accessibility (2SFCA Analysis): spring 2019. Notes: St. Louis City is the area east of the red line. St. Louis County is the area west of the red line. Yellow dots represent meal access points. Darker shades of green represent greater accessibility.

**Figure 4:**
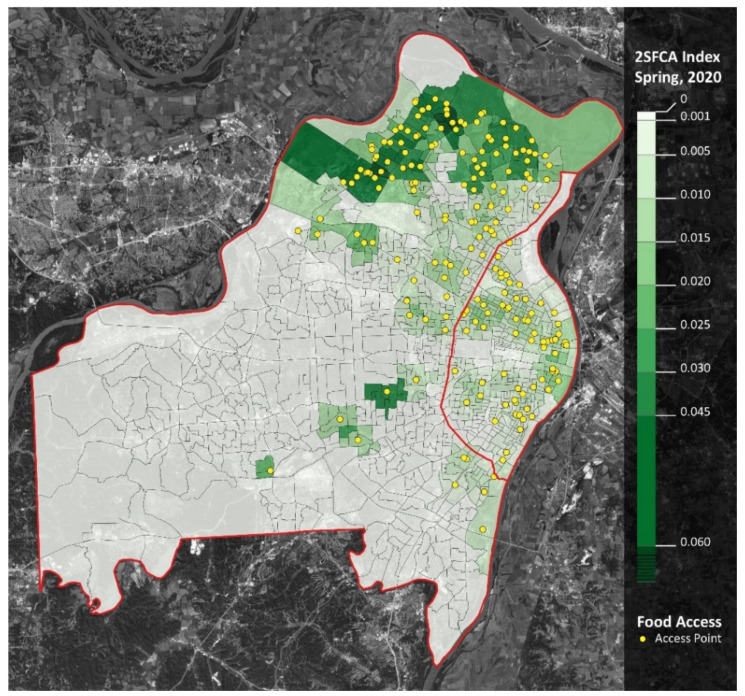
Meal accessibility (2SFCA Analysis): spring 2020. Notes: St. Louis City is the area east of Figure 5 and Figure 6).

However, the opposite trend occurred in the summer: meal accessibility in the summer of 2020 was over four times greater than in the summer of 2019. When comparing school years, accessibility decreased in the western and southern regions of St. Louis County, as well as the southwestern region of St. Louis City. Moreover, accessibility increased in the northern region of St. Louis County (Figure 3 and Figure 4).

### 4.3. How Do Changes in Meal Accessibility during COVID-19 Relate to Race and Socioeconomic Status?

**Figure 6:**
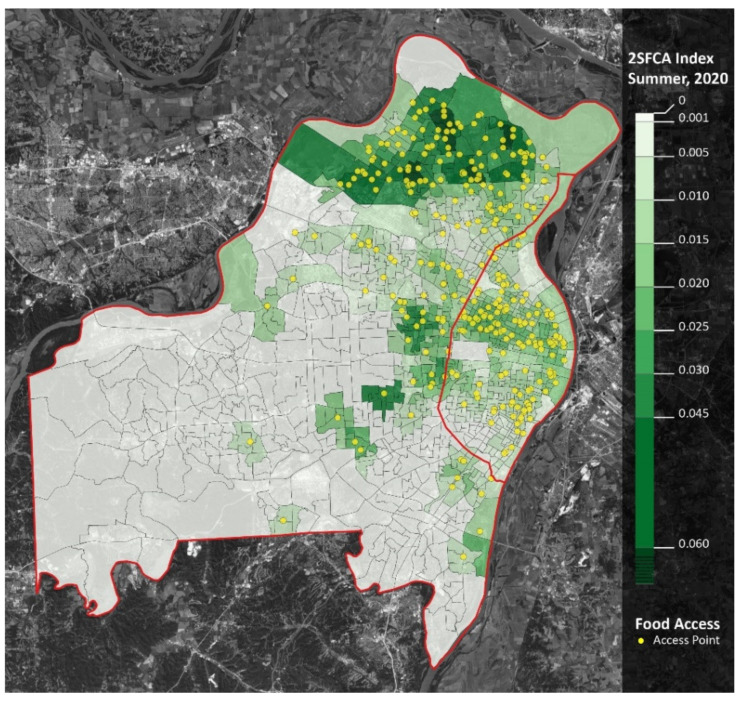
Meal accessibility (2SFCA): summer 2020. Notes: St. Louis City is the area east of the red line. St. Louis County is the area west of the red line. Yellow dots represent meal access points. Darker shades of green represent greater accessibility.

**Figure 7:**
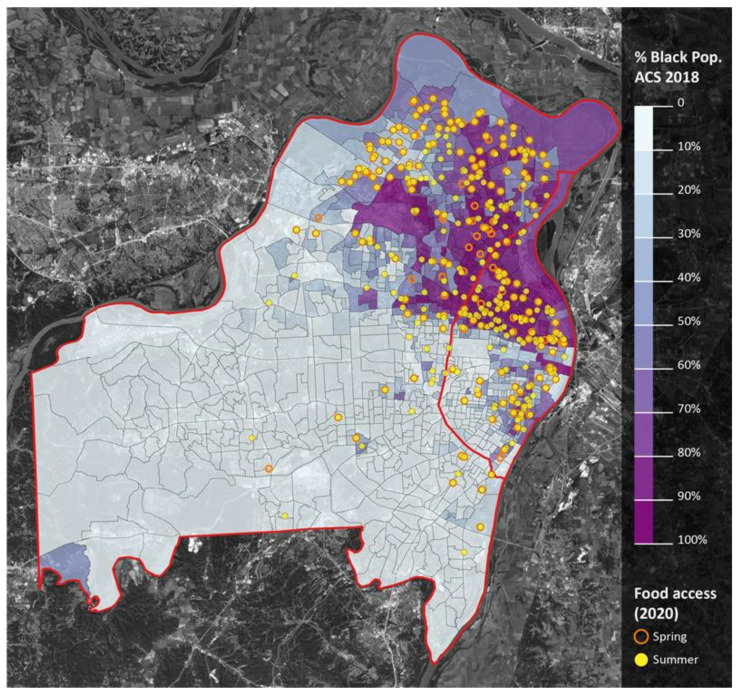
Percent black population and meal access. Notes: St. Louis City is the area east of the red line. St. Louis County is the area west of the red line. Darker shades of purple represent greater proportions of Black residents. Orange circles represent spring meal sites; yellow dots represent summer meal sites.

Finally, when considering the geospatial location of racial minorities, such as the percent of Black families (Figure 7), and how it relates to the geospatial location of expanded summer meal access (Figure 6), it appears that the practices implemented during COVID-19 have improved racial equity in meal access.

**Figure 8:**
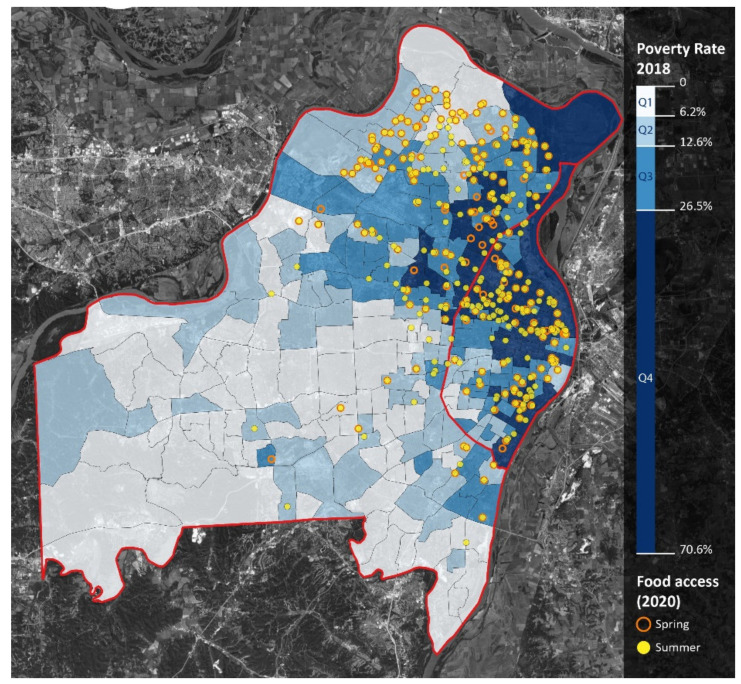
Poverty rate (in quartiles) and meal access. Notes: St. Louis City is the area east of the red line. St. Louis County is the area west of the red line. Darker blue shades represent greater proportions of residents in poverty. Orange circles represent spring meal sites; yellow dots represent summer meal sites.

**Figure 9:**
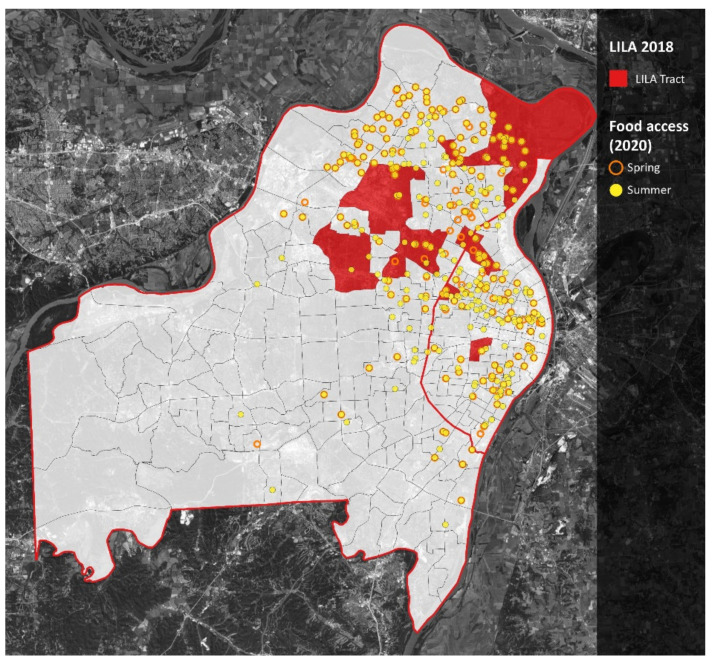
Meal access in low-income and low-access census tracts. Notes: St. Louis City is the area east of the red line. St. Louis County is the area west of the red line. Low-income-low-access (LILA) census tracts are in red. Orange circles represent spring meal sites; yellow dots represent summer meal sites.

This is confirmed when considering that the opposite pattern occurs in the Western (mostly non-Black) regions of St. Louis County and that accessibility did not increase in the southwest (mostly non-Black) region of St. Louis City. Accessibility also increased within and around lower income areas, as well as areas that had lower access (LILA) to food markets (Figure 8 and Figure 9).

## 5. Discussion

This study adds to the emerging literature on school meal accessibility during the COVID-19 pandemic in important ways. While McLoughlin and colleagues demonstrated that meal access during the Spring 2020 semester often served larger areas, higher poverty areas, and areas with greater proportions of minorities in select urban locations [5], more research is needed to understand meal access during the summer months, as well as how meal access changed over time. In filling these gaps, our findings suggest that policy flexibilities implemented during COVID-19 may have improved racial equity in meal access during the pandemic—especially during the summer. However, significantly lower meal access in higher socioeconomic areas suggests a barrier to access for lower-income children living in these areas. Here, low-income students in middle- and upper-income schools—especially in more suburban areas—may face the largest barriers to food access during the pandemic. This finding stresses the important of geographically tailored resource distributions, as suburban areas and the schools within them often have trouble offering services to an increasing population of students from low-income households [25].

While our findings point to the benefits of these policies during the summer months, it is important to note that, traditionally, summer meal program access and participation have been significantly lower than school meal programs offered during the academic year [16], which may contribute to the observed increase in access during the summer. As previous research has demonstrated the promise of summer meals in alleviating child food insecurity [14,15], we present a test case for innovative policies to increase summer meal access. In doing so, it appears that the policy innovations supporting school-year and summer meal programs during COVID-19 provided the infrastructure to increase meal access, which can ultimately mitigate rising levels of food insecurity—a trend that we witnessed during the pandemic [17].

Our results also have clear implications for policy development and program interventions that seek to ensure children have access to food when they are not in school. Specifically, our findings suggest that extending several of the newly implemented policies, such as the SFSP/SSO Area Eligibility Waiver, the Meal Time Waiver, the Non-congregate Feeding Waiver, and the Nationwide Parent/Guardian Meal Pickup Waiver beyond COVID-19 could increase meal access and alleviate child food insecurity during weekends, holidays, and other academic breaks. When considering the relationship between school meals and other academic outcomes [2,10,11,12], these policies may not only improve children’s health, but their academic achievement as well.

Additionally, it is important to note that school meal access is only one of *many* significant geographic barriers that families faced during the COVID-19 pandemic. Recognizing the importance of geography during the pandemic, recent research has used spatial techniques to understand access to testing sites [43], ICU beds [44], and other healthcare resources [45]. Researchers should continue to use geospatial techniques to demonstrate both barriers and opportunities to critical resources—especially when families face new and unprecedented challenges, such as COVID-19.

Finally, given the current availability of data, this study is not without limitations. In particular, data limitations prevented us from examining the number of school meals served at each access point. As a result, we could not assess whether food access points are adequately responding to local demand. For example, as families are able to pick up multiple meals for multiple days, the need for meal access points may be lower during the pandemic. Essentially, it is possible that COVID-19 school meal flexibilities may have improved meal access with fewer access points. Conversely, the demand for summer meals and accompanying access points may have actually been larger than what we found during the summer of 2020, as many summer camps—and other places where meals are typically served—were not open. Nevertheless, if meal access points were operating in equilibrium between supply and demand, then we likely would not have seen the increase in child food insecurity during the Spring 2020 semester, which was widely reported [17]. Furthermore, based on previous research, we know that summer meals were not operating in equilibrium prior to COVID-19, as increases in child food insecurity were consistently reported during the summer months. As additional data become available, future research can complement our examination of geographic access to meal distribution points by considering the number of meals provided, as well as by comparing the observed transition of the school meal ecosystem in St. Louis, Missouri, to other metropolitan areas in the U.S. and other countries.

## 6. Conclusions

The emergence of COVID-19 disrupted the economies, healthcare systems, and educational institutions. While the National School Lunch and Breakfast programs may be effective ways to increase access to school meals in normal times, there were additional challenges in providing meals to students when schools closed during the COVID-19 pandemic. In this paper, we examined the effect of COVID-19 and the resulting policy and program changes to school meal access in St. Louis, Missouri. Specifically, we sought to understand how school meal program access continued during the COVID-19 pandemic as schools changed to virtual formats and families faced a deep and sudden social and economic crisis.

Our findings suggest that federal and state policies and the response of school district administrators maintained a moderate amount of meal accessibility for low-income students in the spring of 2020, while expanding access during the summer of 2020. As previous research demonstrates clear connections between nutrition and educational outcomes, continued access to school meals is paramount for advancing educational and health equity both during and after the COVID-19 pandemic. However, alternative meal access points were not the only policy innovation related to child food insecurity. Thus, future studies should also examine access and utilization of other nutrition supports during COVID-19, including Pandemic-EBT, SNAP, and the USDA Farmers to Families Food Box Program. Prior to COVID-19, many food program policies were restrictive and served as a barrier to participation for low-income families and children—especially during the summer months. While it should not take a global pandemic to come up with new ways of getting meals to low-income families, the responses to COVID-19 offer a blueprint for improving meal access in the future for the families who need it the most.

## Figures and Tables

**Figure 5:**
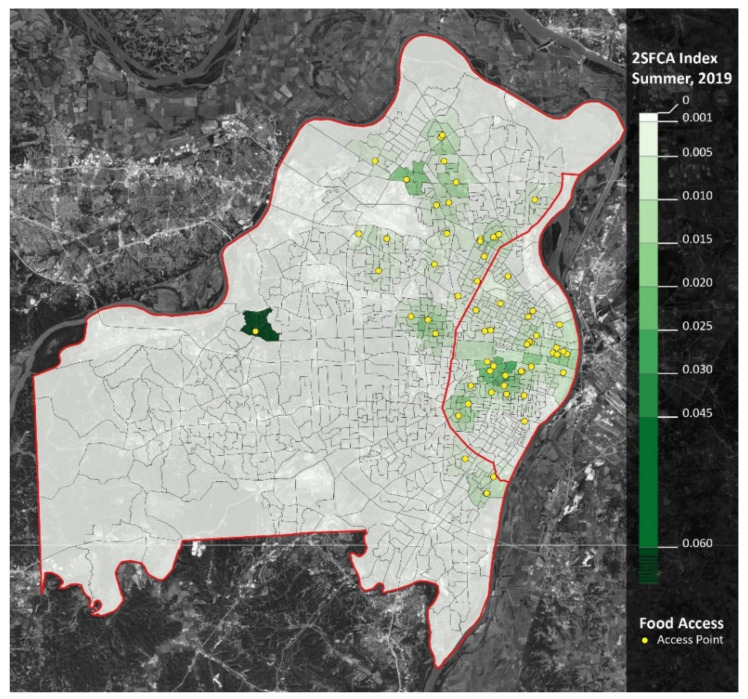
Meal accessibility (2SFCA Analysis): summer 2019. Notes: St. Louis City is the area east of the red line. St. Louis County is the area west of the red line. Yellow dots represent meal access points. Darker shades of green represent greater accessibility.

**Table 1:** Overview of free and reduced-price school meal eligibility in St. Louis, Missouri.

Category	Missouri		Saint Louis	
	Number	Proportion	Number	Proportion
Census Tracts				
Low-Income Tracts	642	46.1%	139	45.6%
Low-Income, Low-Access (LILA) Census Tracts	247	17.7%	31	10.2%
Free and Reduced-Price Lunch (FRPL) Eligibility				
Schools with 0 - 39.9% FRPL Eligible Students	601	27.9%	122	35.6%
Schools with 40 - 62.4% FRPL Elig. Students	746	34.7%	39	11.4%
Schools with 62.5 - 100% FRPL Elig. Students	804	37.4%	182	53.1%

**Table 2:** Food need and access in St. Louis by census tracts.

Average Number of Meal Access Points	Low-Income	Non-Low-Income	LILA	Non-LILA
Saint Louis	Mean (SD)	Mean (SD)	Mean (SD)	Mean (SD)
Spring 2019	1.08 (1.16)	1.22 (1.17)	1.06 (1.06)	1.16 (1.18)
Summer 2019	0.35 (0.89)	0.13 ** (0.40)	0.26 (0.58)	0.23 (0.69)
Spring 2020	0.94 (1.19)	0.45 *** (1.19)	1.19 (1.45)	0.62 (1.17) *
Summer 2020	1.76 (1.68)	0.63 *** (1.35)	2.06 (2.37)	1.04 (1.47) *
Number of Schools	139	165	31	273

Note: *t*-tests were conducted to demonstrate differences between low-income and non-low-income areas, as well as betwen LILA and non-LILA areas; Standard Deviation (SD); * *p* < 0.05; ** *p* < 0.01; *** *p* < 0.001.

**Table 3:** Food need and access by schools.

Category	School Free and Peduce-Priced Lunch (FRPL) Eligibility (Elig.) Categories		
	0–39% FRPL Elig.	40–62.4% FRPL Elig.	62.5–100% FRPL Elig.
	Mean (SD)	Mean (SD)	Mean (SD)
St. Louis			
Average Number of School Meal Access Points (in District)			
Spring 2019	12.5 (9.31)	5.67 *** (3.79)	21.0 ** (31.00)
Summer 2019	0.10 (0.32)	0.33 * (0.58)	6.80 *** (10.70)
Spring 2020	0.69 (0.84)	2.00 ** (2.65)	19.4 *** (28.65)
Summer 2020	1.60 (1.35)	2.67 ** (2.31)	32.5 *** (46.35)
Average Distance to Closest School Meal Access Point (from Schools)			
Spring 2019	0.00 (0.00)	0.00 (0.00)	0.00 (0.00)
Summer 2019	4.64 (2.44)	1.93 *** (1.99)	0.71 *** (0.73)
Spring 2020	3.28 (2.33)	0.50 *** (0.71)	0.38 *** (0.40)
Summer 2020	2.24 (2.02)	0.45 *** (0.63)	0.26 *** (0.29)
Number of Schools	122	39	182

Note: All distance cells for spring 2019 have values of zero because all schools served at least some free and reduced-price meals. *t*-tests were conducted to demonstrate differences between 0–39% FRPL and 40-62.4% FRPL schools, as well as between 0–39% FRPL and 62.5–100% FRPL schools; Standard Deviation (SD); * *p* < 0.05; ** *p* < 0.01; *** *p* < 0.001.

**Table 4:** Accessibility measure.

Category	Two-Step Floating Catchment Area Measure					
	Average	1st Quartile	Median	3rd Quartile	Minimum	Maximum
St. Louis						
Spring 2019	0.0200	0.0057	0.0110	0.0229	0.0000	0.1967
Summer 2019	0.0026	0.0000	0.0000	0.0034	0.0000	0.0909
Spring 2020	0.0068	0.0000	0.0038	0.0088	0.0000	0.0747
Summer 2020	0.0110	0.0000	0.0085	0.0160	0.0000	0.0849

## Data Availability

The authors may provide data upon request.

## References

[1] Auger K.A., Shah S.S., Richardson T., Hartley D., Hall M., Warniment A., Timmons K., Bosse D., Ferris S.A., Brady P.W. (2020). Association Between Statewide School Closure and COVID-19 Incidence and Mortality in the US. JAMA.

[2] Ruffini K. (2021). Universal Access to Free School Meals and Student Achievement: Evidence from the Community Eligibility Provision. J. Hum. Resour..

[3] Van Lancker W., Parolin Z. (2020). COVID-19, school closures, and child poverty: A social crisis in the making. Lancet Public Health.

[4] U.S. Department of Agriculture (2020). National School Lunch Program.

[5] McLoughlin G., McCarthy J., McGuirt J., Singleton C., Dunn C., Gadhoke P. (2020). Addressing Food Insecurity through a Health Equity Lens: A Case Study of Large Urban School Districts during the COVID-19 Pandemic. J. Urban Health.

[6] Radke J., Mu L. (2000). Spatial decompositions, modeling and mapping service regions to predict access to social programs. Geogr. Inf. Sci..

[7] Feeding America Missouri. https://www.feedingamerica.org/hunger-in-america/missouri.

[8] Jyoti D.F., Frongillo E.A., Jones S.J. (2005). Community and International Nutrition. American Society for Nutrition.

[9] Food Research & Action Center Benefits of School Lunch.

[10] Davis W., Musaddiq T. (2018). Estimating the Effects of Subsidized School Meals on Child Health: Evidence from the Community Eligibility Provision in Georgia Schools. SSRN Electron. J..

[11] Gordon N.E., Ruffini K.J. (2019). School nutrition and student discipline: Effects on schoolwide free meals. Educ. Financ. Policy.

[12] Gordanier J., Ozturk O., Williams B., Zhan C. (2020). Free Lunch for All! The Effects of the Community Eligibility Provision on Academic Outcomes. Economics of Education Review.

[13] Billings K. (2020). School Meals and Other Child Nutrition Programs: Background and Funding.

[14] Huang J., Barnidge E., Kim Y. (2015). Children Receiving Free or Reduced-Price School Lunch Have Higher Food Insufficiency Rates in Summer. J. Nutr..

[15] Kuhn M.A. (2018). Who feels the calorie crunch and when? The impact of school meals on cyclical food insecurity. J. Public Econ..

[16] Hayes C., Rosso R., FitzSimons C. (2019). Hunger Doesn’t Take a Vacation.

[17] Ahn S., Norwood F.B. (2020). Measuring Food Insecurity during the COVID-19 Pandemic of Spring 2020. Appl. Econ. Perspect. Policy.

[18] Miller D.P. (2016). Accessibility of summer meals and the food insecurity of low-income households with children. Public Health Nutr..

[19] Wilkerson R.L., Khalfe D., Krey K. Associations between Neighborhoods and Summer Meals Sites: Measuring Access to Federal Summer Meals Programs.

[20] Aussenberg R., Billings K. (2020). USDA Domestic Food Assistance Programs’ Response to COVID-19: P.L. 116–127, P.L. 116- 136, and Related Efforts.

[21] Barton T. (2020). COVID-19’s Impact on Healthy Meals for Low-Income Families.

[22] U.S. Department of Agriculture (2020). FNS Program Guidance on Human Pandemic Response.

[23] Sonnino R. (2016). The new geography of food security: Exploring the potential of urban food strategies. Geogr. J..

[24] Walker R.E., Keane C.R., Burke J.G. (2010). Disparities and access to healthy food in the United States: A review of food deserts literature. Health Place.

[25] Allard S., Roth B. (2010). Strained Suburbs: The Social Service Challenges of Rising Suburban Poverty.

[26] National Center for Education Statistics (2018). Number and percentage of public school students eligible for free or re-duced-price lunch, by state: Selected years, 2000–01 through 2018–19. https://nces.ed.gov/programs/digest/d20/tables/dt20_204.10.asp.

[27] United States Census Bureau (2018). American Community Survey 2018 Data Profiles.

[28] Chetty R., Friedman J., Hendren N., Stepner M. (2020). The Opportunity Insights Economic Tracker: Supporting the Recovery from COVID-19. Opportunity Insights. tracktherecovery.org.

[29] Missouri Department of Elementary and Secondary Education (2020). Coronavirus (COVID-19) Information.

[30] Charreire H., Casey R., Salze P., Simon C., Chaix B., Banos A., Badariotti D., Weber C., Oppert J.M. (2010). Measuring the food environment using geographical information systems: A methodological review. Public Health Nutr..

[31] Chen X. (2017). Take the edge off: A hybrid geographic food access measure. Appl. Geogr..

[32] Chen X. (2019). Enhancing the Two-Step Floating Catchment Area Model for Community Food Access Mapping: Case of the Supplemental Nutrition Assistance Program. Prof. Geogr..

[33] Rhone A., Ver Ploeg M., Williams R., Breneman V. (2019). Understanding Low-Income and Low-Access Census Tracts Across the Nation: Subnational and Subpopulation Estimates of Access to Healthy Food.

[34] Missouri Department of Health and Senior Services (2020). Missouri Coronavirus GIS Hub. https://missouri-coronavirus-gis-hub-mophep.hub.arcgis.com/app/6f227b5862534b92a6d6ef91c9cbb903.

[35] Missouri Spatial Data Information Service Welcome to MSDIS. http://msdis.missouri.edu/.

[36] Rhone A., Ver Ploeg M., Dicken C., Williams R., Breneman V. (2017). Low-Income and Low-Supermarket-Access Census Tracts, 2010–2015.

[37] Despard M., Grinstein-Weiss M., Chun Y., Roll S. (2020). COVID-19 Job and Income Loss Leading to more Hunger and Financial Hardship.

[38] Rahkovsky I., Snyder S. (2015). Food Choices and Store Proximity.

[39] St. Louis University PRiME Center. Missouri Education Data. https://www.sluprime.org/education-data.

[40] Missouri Department of Elementary and Secondary Education Community Eligibility Provision. https://dese.mo.gov/financial-admin-services/food-nutrition-services/community-eligibility-provision-cep.

[41] Ragain T., Ritter S., Wilbers Cavender L., Ferris D., Rothman S., Hsu Y. (2021). Missouri School Breakfast Report: School Year 2018–2019.

[42] US Department of Education (2015). The Community Eligibility Provision and Selected Requirements under Title I, Part A of the Elementary and Secondary Education Act of 1965, as Amended.

[43] Tao R., Downs J., Beckie T.M., Chen Y., McNelley W. (2020). Examining spatial accessibility to COVID-19 testing sites in Florida. Ann. GIS.

[44] Kim K., Ghorbanzadeh M., Horner M.W., Ozguven E.E. (2021). Identifying areas of potential critical healthcare short ages: A case study of spatial accessibility to ICU beds during the COVID-19 pandemic in Florida. Transp. Policy.

[45] Kang J.Y., Michels A., Lyu F., Wang S., Agbodo N., Freeman V.L., Wang S. (2020). Rapidly measuring spatial accessibility of COVID-19 healthcare resources: A case study of Illinois, USA. Int. J. Health Geogr..

